# Deficiency of endothelial FGFR1 alleviates hyperoxia-induced bronchopulmonary dysplasia in neonatal mice

**DOI:** 10.3389/fphar.2022.1039103

**Published:** 2022-11-18

**Authors:** Yanrong Long, Hongbin Chen, Junchao Deng, Junjie Ning, Pengbo Yang, Lina Qiao, Zhongwei Cao

**Affiliations:** Key Laboratory of Birth Defects of MOE, State Key Laboratory of Biotherapy, West China Second University Hospital, Sichuan University, Chengdu, China

**Keywords:** bronchopulmonary dysplasia (BPD), hyperoxia, endothelial cells (ECs), FGFR1, neonatal

## Abstract

Disrupted neonatal lung angiogenesis and alveologenesis often give rise to bronchopulmonary dysplasia (BPD), the most common chronic lung disease in children. Hyperoxia-induced pulmonary vascular and alveolar damage in premature infants is one of the most common and frequent factors contributing to BPD. The purpose of the present study was to explore the key molecules and the underlying mechanisms in hyperoxia-induced lung injury in neonatal mice and to provide a new strategy for the treatment of BPD. In this work, we reported that hyperoxia decreased the proportion of endothelial cells (ECs) in the lungs of neonatal mice. In hyperoxic lung ECs of neonatal mice, we detected upregulated fibroblast growth factor receptor 1 (FGFR1) expression, accompanied by upregulation of the classic downstream signaling pathway of activated FGFR1, including the ERK/MAPK signaling pathway and PI3K-Akt signaling pathway. Specific deletion of *Fgfr1* in the ECs of neonatal mice protected the lungs from hyperoxia-induced lung injury, with improved angiogenesis, alveologenesis and respiratory metrics. Intriguingly, the increased *Fgfr1* expression was mainly attributed to aerosol capillary endothelial (aCap) cells rather than general capillary endothelial (gCap) cells. Deletion of endothelial *Fgfr1* increased the expression of gCap cell markers but decreased the expression of aCap cell markers. Additionally, inhibition of FGFR1 by an FGFR1 inhibitor improved alveologenesis and respiratory metrics. In summary, this study suggests that in neonatal mice, hyperoxia increases the expression of endothelial FGFR1 in lung ECs and that deficiency of endothelial *Fgfr1* can ameliorate hyperoxia-induced BPD. These data suggest that FGFR1 may be a potential therapeutic target for BPD, which will provide a new strategy for the prevention and treatment of BPD.

## Introduction

In neonates, especially premature infants, the premature developing lungs are often susceptible to damage from many factors, such as hyperoxia, which often results in bronchopulmonary dysplasia (BPD). It is the most common chronic lung disease in preterm birth and is mainly characterized by simplified vascularization and alveolarization and even fibrosis in severe cases (Northway et al., 1967; Thébaud et al., 2019; Sahni and Mowes, 2022). As a consequence of BPD, residual pulmonary dysfunction and cardiovascular sequelae in adolescence and adulthood may occur (Islam et al., 2015; DeMauro, 2018; Tracy and Berkelhamer, 2019).

The development of the mammalian lung is broadly divided into two phases: prenatal phase and postnatal phase. During the prenatal phase, the core lung structure containing the branching conducting airways with their attendant vasculature develops, while the alveolarization and the maturation of pulmonary microvasculature are mainly completed at the postnatal phase (Warburton et al., 2005; Chang et al., 2013; Silva et al., 2015; Schittny, 2017). The two phases of lung development are intimately coordinated, and being disturbed at any phase may provoke lung disease (Chang et al., 2013). BPD is thought to be the result of an abnormal reparative response to both antenatal injury and repetitive postnatal injury to the developing lungs (Thébaud et al., 2019). Endothelial cells (ECs) play an important role in angiogenesis, which fundamentally contributes to lung development, homeostasis, and injury repair (Ding et al., 2011; Miller and Sewell-Loftin, 2021; Filippini et al., 2022; Zhou et al., 2022). There is complicated reciprocal signaling between ECs and epithelial cells, which regulates the formation of an extensive capillary network to support lung development (Ren et al., 2019).

General capillary endothelial cells (gCap cells) and aerosol capillary endothelial cells (aCap cells) are two subpopulations of ECs that make up the alveolar endothelium. gCap cells are specialized to regulate vasomotor tone and are considered as stem/progenitor cells in capillary homeostasis and repair, while aCap cells are specialized for gas exchange and trafficking of leukocytes (Gillich et al., 2020). Prior studies showed that adoptively transferred *c-kit* ^+^ ECs (gCap cells) increased lung angiogenesis and prevented alveolar simplification in neonatal mice exposed to hyperoxia (Ren et al., 2019). Furthermore, it was previously reported that after acute lung injury, *Car4*-high ECs (aCap cells) are preferentially localized in regenerating regions of the alveolus and contribute to alveolar revascularization (Niethamer et al., 2020). Although previous studies have reported that ECs and their subpopulations play an important role in impaired lung repair, the underlying mechanisms by which ECs and their subpopulations act in BPD are unclear.

Here, we detected the alterations in ECs and their subpopulations in hyperoxia-impaired neonatal lungs by scRNA-seq and RNA-seq. We found that endothelial fibroblast growth factor receptor 1 (FGFR1), a member of the fibroblast growth factor receptor (FGFR) family, may play an important role in hyperoxia-induced BPD. FGF/FGFR signaling was reported to be crucial for many physiological activities, such as embryonic development, organogenesis, and tissue maintenance (Dow and deVere White, 2000; Bates, 2011; Goetz and Mohammadi, 2013; Yu et al., 2017; Kurowski et al., 2019). Some scholars believe that FGF/FGFR signaling plays a proangiogenic role in physiological or pathological conditions (Yanagisawa-Miwa et al., 1992; Deng et al., 1994; Compagni et al., 2000; De Smet et al., 2014). In addition, it was previously found that FGFR1 is the key inhibitor of endothelial-to-mesenchymal transition (EndMT) and is important in combating EndMT-associated fibrotic disorders (Li et al., 2017). Nevertheless, scholars have reported that FGF/FGFR signaling is not essential for angiogenesis and injured vessel repair (Ortega et al., 1998; Zhou et al., 1998; Miller et al., 2000; Ong et al., 2000). Exaggerated FGF2/FGFR1 signaling caused by SUMOylation-defective mutation of FGFR1 suppressed VEGFA/VEGFR2 signaling and the angiogenic capabilities of ECs (Zhu et al., 2022). Additionally, it was previously reported that continuous activation of endothelial FGFR1 can promote the formation of profibrotic vascular niche, which would facilitate fibrosis in chronic liver injury (Ding et al., 2014). Moreover, there is another point of view that endothelial FGFR1 is necessary for pathological neovascularization after injury but not for physiological vascular development and vascular homeostasis (Oladipupo et al., 2014; House et al., 2016). These studies indicated that FGFR1 performs different and potentially important functions in different physiological situations. In this work, we found that hyperoxia increased the expression of endothelial FGFR1, which indicated that endothelial FGFR1 may be a key regulatory molecules in hyperoxia-induced BPD. However, the role of FGFR1 in hyperoxia-induced BPD have not been reported. Therefore, it is meaningful to throw light on this question.

To mimic lung injuries in patients with BPD, firstly, wild-type (WT) neonatal mice were exposed to hyperoxia (80% O_2_) to induce a BPD-like lung phenotype, which was suggested to be an ideal model to identify and study pivotal developmental steps of lung injury and repair (Nardiello et al., 2017; Surate Solaligue et al., 2017; Ito et al., 2022). Mice reared in room air were used as controls. Consistent with previous studies (Nardiello et al., 2017; Ito et al., 2022). We observed that hyperoxia exposure resulted in abnormal angiogenesis and disrupted alveologenesis, the prominent features of BPD, in neonatal mice. Next, single-cell RNA sequencing (scRNA-seq) was performed to investigate the cellular and molecular changes in hyperoxia-damaged lungs. The results of scRNA-seq analysis suggested that hyperoxia decreased the proportion of ECs in the lungs of neonatal mice. Then, we employed whole transcriptome sequencing (RNA-seq) to further explore and confirm the molecular changes in ECs during the occurrence and development of hyperoxia-based BPD. We found that hyperoxia increased the expression of endothelial FGFR1. Therefore, using the same hyperoxia procedure, we used a genetically modified mouse in which *Fgfr1* was conditionally deleted specifically in ECs (*Fgfr1* ^iΔEC^/^iΔEC^) to investigate the role of FGFR1 in hyperoxia-induced lung injury and repair.

## Materials and methods

### Mice and neonatal hyperoxia

The WT C57BL/6J mice were obtained from the Model Animal Research Center of Nanjing University. Floxed *Fgfr1* (*Fgfr1* ^fl/fl^) C57BL/6J mice were generously provided by Dr. Shahin Rafii. Mice expressing EC-specific *VE-Cadherin-(PAC)-Cre* ^ERT2^ were obtained from Taconic Biosciences. *Fgfr1* ^iΔEC/iΔEC^ mice were generated by crossing *Fgfr1* ^fl/fl^ mice with *VE-Cadherin-(PAC)-Cre* ^ERT2^ mice. From the day of birth (P0) to postnatal day (P)2, *Fgfr1* ^iΔEC/iΔEC^ mice were intraperitoneally treated with tamoxifen (50 µg/mouse/day) to induce endothelial-specific deletion of *Fgfr1*. The neonatal hyperoxia exposure was performed to develop the BPD mouse model according to previously described protocols (Silva et al., 2015; Nardiello et al., 2017; Cheon et al., 2018; Willis et al., 2018). At P0, neonatal mouse pups delivered on the same day were randomly divided into equal-sized litters around six pups nursed by each dam. Then, following randomization, mouse cages were either maintained in room air (21% O_2_) or in hyperoxia (80% O_2_) from P0 to P14. Lungs of mouse pups were harvested for testing at P14. The hyperoxic environment was maintained in sealed Plexiglas chambers, which contained sodium bicarbonate as an odor adsorbent and sodium hydroxide as an H_2_O adsorbent. The chamber contained a continuous oxygen monitoring system (ProOX-100HE, TOW-INT TECH) and was attached to a medical oxygen source. To avoid oxygen toxicity, nursing dams were rotated between normoxic and hyperoxic groups every 24 h. All mice were housed under SPF conditions (12/12 h light/dark cycle, 50%–70% relative humidity, temperature was maintained between 26°C and 28°C) and with access to *ad libitum* feeding. All mouse pups were euthanized at P14 to harvest lungs for testing.

### FGFR1 inhibitor (AZD4547) injection

The FGFR1 inhibitor (AZD4547) was generously provided by Dr. Tinghong Ye. For *in vivo* efficacy studies, neonatal mice were randomly divided into three groups: room air + vehicle, hyperoxia + vehicle or hyperoxia + inhibitor. From P0 to P5, neonatal mice were daily treated with vehicle or inhibitor (5 μg inhibitor dissolved in 20 μL vehicle: 5% DMSO + 40% PEG 400 + 55% saline) by intragastric injection through the abdominal wall according the dose used in a previously reported study (Gudernova et al., 2016). Each mouse was injected with 20 μL vehicle or 5 μg inhibitor/20 μL vehicle by using a 50-ml Hamilton microsyringe. The hyperoxia exposure and feeding management protocols were the same as those described above. All mouse pups were euthanized at P14 to harvest lungs for testing.

### Histology

At P14, the lungs were inflated with 20 ml 4% paraformaldehyde in PBS under constant pressure of 20 cm H_2_O and allowed to fix for 24 h. After being fixed with 4% paraformaldehyde, the tissues were dehydrated with an ethanol series, embedded in paraffin and sectioned. Hematoxylin and eosin (H&E) staining was performed for tissue morphology examination and mean linear intercept (MLI) and radial alveolar count (RAC) measurement. Analysis of sections was recorded with an Olympus BX51 microscope (Olympus America). The MLI and RAC were measured following a previously described protocol (Cooney and Thurlbeck, 1982; Ashour et al., 2006). For each animal, one lung sections was prepared on a slide, and 5 randomized microscopic fields in each section were selected for MLI or RAC measurement. The average value of the 5 microscopic fields was calculated to represent the results for individual mouse.

### Immunofluorescence

At P14, after the mice being sacrificed, the lungs were inflated with 20 ml PBS under constant pressure of 20 cm H_2_O from the left atrium. Then, lung tissues were embedded in Optimum Cutting Temperature (OCT) and made into cryosections. Briefly, at first, each lung was slowly filled with 50 μL OCT:PBS (1:1) mixture from the trachea. Secondly, the lungs were removed from the chest cavity and placed into tissue embedding cassettes with the maximum side down and carefully filled with OCT into the cassettes. Placed the filled cassettes containing embedded tissues onto the flat surface and quickly placed them into a -80°C refrigerator to freeze the embedded tissue. Next, cryosections were made from the frozen embedded tissue for immunofluorescence (IF) analysis. Six-millimeter-thick tissue cryosections were blocked (5% donkey serum/0.3% Triton X-100) and incubated in primary antibodies at 4°C overnight (anti-VE-cadherin antibody, R&D Systems, #AF1002). After incubation with primary antibodies, slides were washed three times with PBS, followed by incubation in fluorophore-conjugated secondary antibodies (Jackson ImmunoResearch), and nuclear staining was carried out with DAPI by using Prolong Gold Anti-fade Reagent (Invitrogen). Finally, fluorescent images were recorded on an AxioVert LSM980 confocal microscope (Zeiss) for analysis. ImageJ (version: 2.0.0-rc-69/1.52p) was used for fluorescent image analysis following a previously described protocol (Zhou et al., 2021). For each animal, one lung section was prepared on a slide and 5 randomized microscopic fields in each section were selected for fluorescence analysis. The average value of the 5 microscopic fields was calculated to represent the results for individual mouse.

### Respiratory metrics testing

The respiratory metrics were quantified by whole-body plethysmography (WBP, DSI Buxco) at P14. Mice were examined in a calibrated WBP chamber, and the manufacturer software was used to calculate respiratory measures. After the respiratory data stabilized, data were recorded for 5 min per mouse. The average was calculated to represent the results for individual mouse.

### Single-cell RNA sequencing

Single-cell suspension was prepared following a previously described protocol (Zepp et al., 2017). Briefly, lungs were perfused with PBS, minced with scissors and digested in an enzymatic mixture (dispase II: 200 μg/ml, collagenase I: 200 μg/ml, DNase I: 40 μg/ml) for 35 min at 37°C. Following enzymatic digestion, the samples were filtered, and red blood cells were lysed. Then, single cells were resuspended in MACS buffer (DPBS + 0.1% BSA + 2 mM EDTA). Ensure cell viability exceeded 80% as determined by AO/PI reagent staining. The following sequencing steps were performed by 10X Genomics. The scRNA-seq profiles of 17,320 cells from 16 normoxic and hyperoxic mouse lungs were generated with 150 G sequencing depth. Single-cell suspensions were loaded into 10x Chromium to capture 10,000 single cells according to the manufacturer’s instructions of the 10X Genomics Chromium Single-Cell 3′ Kit (V3). Using microfluidic techniques, gel beads with barcodes and primers were wrapped in oil droplets (GEMs) with individual cells. Then the gel beads were dissolved and the cells were lysed to release the mRNA. cDNA with barcode and UMI information for sequencing was generated by reverse transcription of the mRNA. The cDNA amplification and library construction steps were performed according to the standard protocol. Libraries were sequenced on an Illumina NovaSeq 6,000 sequencing system (paired-end multiplexing run, 150 bp). Sequencing results were converted to FASTQ format using Illumina bcl2fastq software (version 2.20). Cell Ranger pipelin (version 6.1.1) was used for sample demultiplexing, barcode processing and gene counting, and scRNA-seq data were aligned to the reference genome (Transcriptome: mm10–1.2.0). Seurat (Version 4.0) was used for dimensional reduction, clustering and analysis of scRNA-seq data. Each group contained eight mice mixed into one sample for scRNA-seq.

### iDISCO (ace) procedure

The iDISCO (ace) procedure was performed according to the previously described protocol (Liu et al., 2020). The VE-cadherin antibody conjugated with Alexa Fluor™ 647 (VE-cadherin antibody, Biolegend, #138002. Alexa Fluor™ 647 dye, Thermo Fisher, #A20006) was diluted 1:1000 for use. After immunolabeling with Alexa Fluor dye-conjugated VE-Cadherin antibody, the tissues were washed directly with PBS/0.1% Tween 20/heparin (10 μg/ml) at room temperature for 24 h following the protocol described. The lung tissues processed by the iDISCO (ace) procedure were imaged on an Andor Dragonfly 200 Confocal Imaging System, with a ×10 objective, a step size of 8μm, 250 ms exposure time. About 1200-μm-thick optical stack of signal were acquired for each lung tissue. Imaris (version 9.9) was used to reconstruct the image stacks obtained from the confocal imaging to perform whole-tissue 3D assessment of the vasculature. AngioTool (version 0.6a) was used for vessels percentage area and total number of junctions analysis (Zudaire et al., 2011).

### Vascular leakage experiment

After being euthanasiaed, the mice were treated with FITC-dextran (Sigma, #R9379-250 MG, 50 mg/ml, 25 μL/mouse) via lateral tail vein injection. 30 min after FITC-dextran injection, the mice were sacrificed to harvest the lung tissues. After the mice being sacrificed, skipping the step of inflating with PBS, lung tissues were removed from the chest cavity and directly embedded in OCT according to the protocol described above and then made into cryosections. Nuclear staining with DAPI was performed according to the IF protocol described above. Finally, the tissue sections were imaged on an AxioVert LSM980 confocal microscope (Zeiss) for analysis. The fluorescence analysis protocol was the same as that for IF described above.

### Whole transcriptome sequencing (RNA-seq)

Single-cell suspensions were prepared as described above. Next, the single-cell suspension was incubated with CD45-coated beads for 30 min at 4°C, the bead-bound cells were removed using a magnet, and the unbound cell suspension was collected, followed by incubation with CD31-coated beads for 30 min at 4°C. The CD31-coated bead-bound cells were collected and washed 5 times with PBS/0.1% BSA/2 mM EDTA. Purified Rat Anti-Mouse CD31 (#553370) and Purified Rat Anti-Mouse CD45 (#553076) were obtained from BD Biosciences, and Dynabeads Sheep anti-Rat IgG (#11035) was obtained from ThermoFisher Scientific. Total RNA was prepared from the collected ECs using TRIzol (ThermoFisher, #15596018) following the manufacturer’s procedure. Then the 2 × 150 bp paired-end sequencing (PE150) was performed on an Illumina NovaseqTM 6,000 following the vendor’s recommended protocol. Count data for genes were analyzed in R using the DESeq2 software package (version 1.30.1).

### Quantitative RT‒PCR

ECs were isolated as described above. Total RNA was prepared from lung ECs using TRIzol following the manufacturer’s procedure. Complementary DNAs (cDNA) were synthesized from the total RNA using the PrimeScript RT Reagent Kit (TaKaRa, #RR0447A), and qPCR was performed on a CFX96 Real-Time PCR Detection System (Bio-Rad). Gene mRNA expression was quantified using SYBR green technology, with *β-actin* and *Gapdh* used as internal controls. Primer sequences are available upon request.

### Western blot

Protein was extracted from lung ECs isolated as described above. Equal amounts of protein were separated in a 10% gel and transferred to nitrocellulose membranes. The membranes were incubated overnight with primary anti-FGFR1 antibody (1:1000; Cell Signaling Technology, #9740). Gapdh (1:1000, Santa Cruz #sc-32233) was used as an internal control. Signals were detected using corresponding horseradish peroxidase-conjugated secondary antibodies (1:5000, Abcam, #ab205719, #ab6721) and enhanced chemiluminescence (ThermoFisher, #34095).

### Quantification and statistical analysis

Calculations were carried out with the Excel and Prism nine software packages (Version 9.1.1). One-way ANOVA followed by Tukey’s test was employed to determine statistical significance. All data are presented as means ± standard error of means (SEMs). Error bar shows SEMs and center shows means. *p* < 0.05 was considered statistically significant.

## Results

### Neonatal hyperoxic lung injury results in disrupted alveolar development, respiratory dysfunction and abnormal vascular development

**FIGURE 1:**
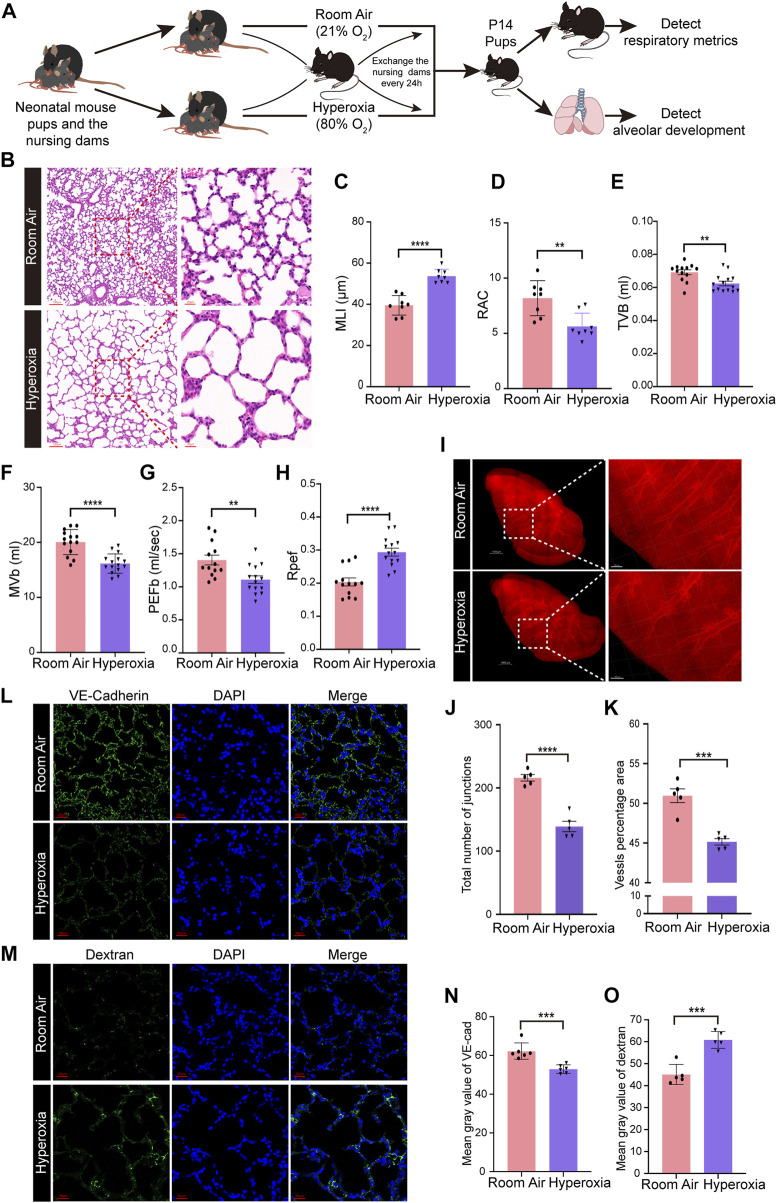
Hyperoxia disrupted angiogenesis and alveologenesis and resulted in respiratory dysfunction inneonatal mice. **(A)** Approach to develop a mouse model of BPD. Mouse pups were exposed to room air (21% O_2_) or hyperoxia (80% O_2_) from the day of birth (P0) to postnatal days (P)14. After measuring the respiratory metrics at P14, lungs were harvested for detection. **(B)** Representative images of H&E-stained lungs. The left panel shows low-magnification (scale bar = 100 μm) images, and the right panel shows higher-magnification (scale bar = 20 μm) images. **(C,D)** Quantification of MLI (C) and RAC (D) based on the data in (B). Data are shown as the means ± SEMs. *n* = 8 per group. **p < 0.01, ****p < 0.0001. **(E–H)** Results of respiratory metrics measurement. Data are shown as the means ± SEMs. *n* = 13 or 14 per group. **p < 0.01, ****p < 0.0001. **(I–K)** Whole-tissue 3D assessment of vasculature in normal and hyperoxia-impaired lungs. 3D volume fluorescence images of VE-cadherin (VE-Cad)-stained lungs. The left panel shows low-magnification (scale bar = 1000 μm) images, and the right panel shows higher-magnification (scale bar = 200 μm) images (I). Quantification of the total number of junctions (J) and vessels percentage area (K) based on the data in (I). Data are shown as means ± SEMs. n = 5 per group. ***p < 0.001, ****p < 0.0001. (**L**) Representative images of IF staining for VE-Cad in lung sections. Magnification: ×40. Scale bar = 20 μm. **(N)** Mean gray value of VE-Cad based on the data in (L). Data are shown as means ± SEMs. *n* = 6 per group. ***p < 0.001. **(M)** Representative images of FITC-dextran leakage from mice reared in room air or hyperoxia. Magnification: ×40. Scale bar = 20 μm. **(O)** Mean gray value of dextran based on the data in (M). Data are shown as means ± SEMs. *n* = 5 per group. ***p < 0.001.

WT neonatal mice were exposed to hyperoxia (80% O_2_) for 14 days to develop a mouse model of BPD, while the mice living in room air were used as controls (Figure 1A). Hyperoxia interrupted lung development in neonatal mice, resulting in enlarged alveoli and reduced alveolar numbers (Figures 1B–D). Mice from the hyperoxia group had an increased mean linear intercept (MLI) (Figure 1C) and a decreased radial alveolar count (RAC) (Figure 1D). Compared with mice from the room air group. The results of respiratory metrics measurement showed a significant decrease in minute ventilation (MVb), tidal volume breathing (TVb) and peak expiratory flow (PEFb) in hyperoxic mice compared to normoxic mice (Figures 1E–G), but a significant increase in Rpef, the ratio of time to peak expiratory flow (PEF) relative to total expiratory time (Te) (Figure 1H). These data mainly show that hyperoxia provokes disrupted alveologenesis, a prominent phenotype of BPD, as well as respiratory dysfunction in neonatal mice.

Next, we detected the influence of hyperoxia on pulmonary vascular development. The iDISCO (ace) tissue clearing procedure followed by immunofluorescence staining was employed to assess the development of pulmonary vasculature. The results showed that hyperoxia-exposed mice had reduced vascular branching and density (Figures 1I–K). The total number of junctions and vessels percentage area were reduced significantly in hyperoxic lungs (Figures 1J,K). Moreover, the results of IF staining showed that the expression of vascular endothelial cadherin (VE-Cad), a marker of endothelial cells, was lower in lungs developed in hyperoxia than in lungs developed in normoxia (Figures 1L, N). We further performed a FITC-dextran leakage experiment to evaluate the barrier function of blood vessels, which indicated that hyperoxia increased vascular leakage (Figures 1M, O). These results confirmed that hyperoxia results in abnormal vascular development, which thought to be one of the main mechanisms contributing to disrupted alveologenesis.

### Hyperoxia induces ECs loss and upregulates the expression of endothelial FGFR1 and the classic FGFR1 signaling pathways in ECs

**FIGURE 2:**
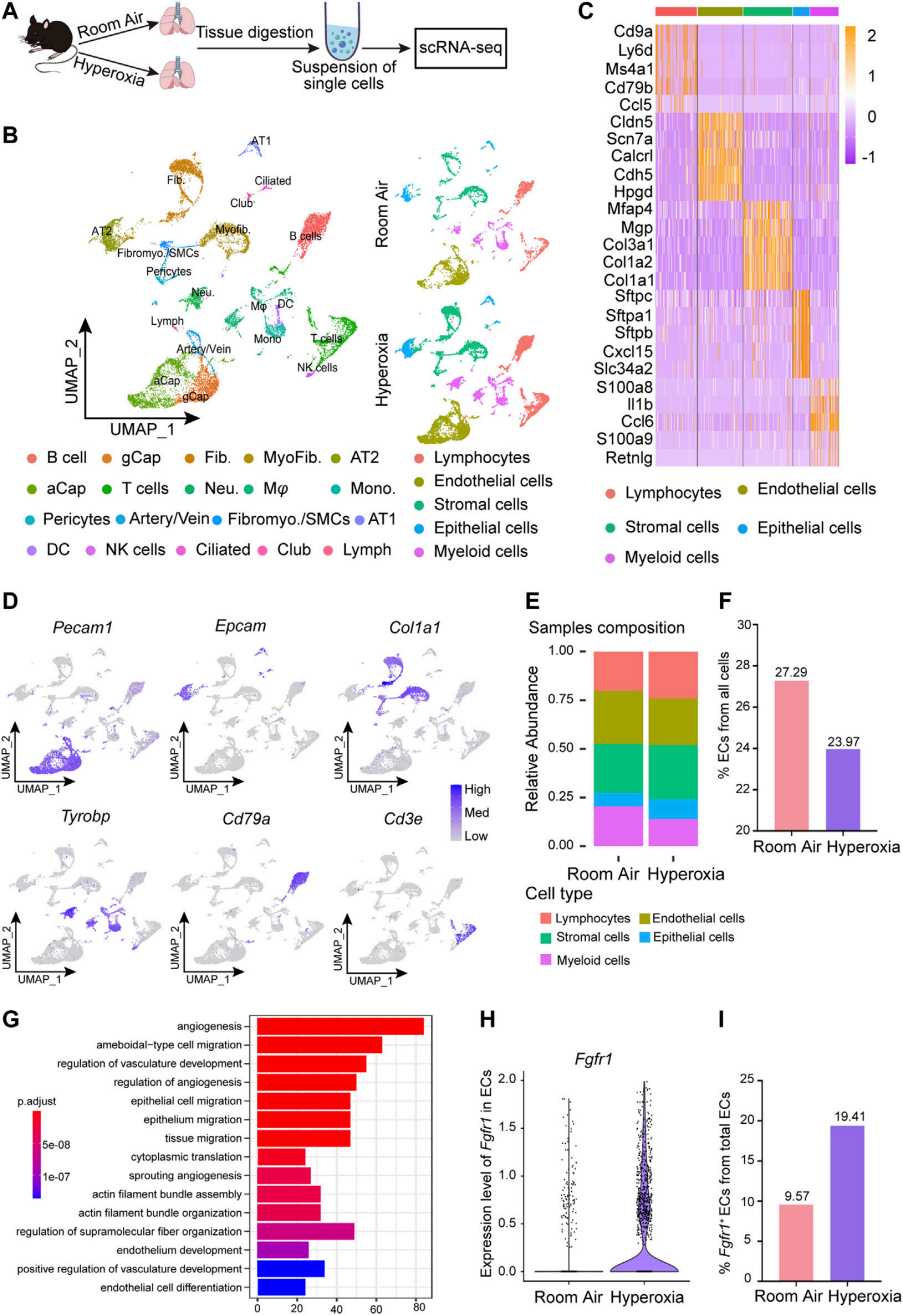
scRNA-seq analysis of lungs from normoxia- and hyperoxia-reared mice. **(A)** Approach to generate a single-cell atlas. **(B)** UMAP plot of all scRNA-seq data, showing a total of 19 distinct cell types corresponding to 5 major cell groups. Cell populations are colored as indicated by the legend. **(C)** Heatmap of the top 5 most differentially expressed genes across 5 major cell types. The intensity of expression is indicated as specified by the color legend. **(D)** Feature plots showing the expression of principal identifiers of epithelial cells, endothelial cells, stromal cells, myeloid cells, lymphocytes (B cells) and lymphocyte (T cells) populations. **(E)** Cellular compositions are colored as indicated by the legend in normal and hyperoxia-impaired lungs. **(F)** The relative proportion of endothelial cells from all cells in normal and hyperoxia-impaired lungs. **(G)** Hyperoxia-impacted signaling pathways in ECs, as identified by GO enrichment analysis of biological processes. **(H)** Violin plot showing the expression of *Fgfr1* in ECs. **(I)** *Fgfr1* ^+^ ECs in total ECs.

To investigate the cellular and molecular changes resulting from neonatal lung injury induced by hyperoxia, scRNA-seq was performed on a 10X genomics platform to generate scRNA-seq profiles of WT mice reared in normoxia or hyperoxia (Figure 2A). Five major cell lineages (lymphocytes, endothelial cells, stromal cells, epithelial cells and myeloid cells) corresponded by 19 cell clusters were identified by the expression of marker genes (Figures 2B–D; Table 1). We noted that the frequency of ECs decreased in the hyperoxia-impaired lungs (Figures 2E,F), which is consistent with the results obtained by analyzing the raw data from Thébaud who generated scRNA-seq profiles of 66,200 cells from normally (21% O_2_-exposed from P0-P14) and aberrantly (85% O_2_-exposed from P0-P14) developing mouse lungs at P14 (Supplementary Figure S1D,E). Gene ontology (GO) enrichment analysis of the endothelial cell population suggested that the pathways associated with angiogenesis were impacted by hyperoxia exposure (Figure 2G). Furthermore, the GO enrichment analysis showed that hyperoxia impacted the signaling pathways associated with epithelial cell migration (Figure 2G). In addition, the results of scRNA-seq analysis showed an increase in the expression of *Fgfr1* in ECs from hyperoxic lungs (Figure 2H) and that the proportion of *Fgfr1*-positive (*Fgfr1* ^+^) ECs increased in hyperoxic lungs (Figure 2I).

**TABLE 1:** Identified cell populations.

Abbreviation	Cell type	Abbreviation	Cell type
aCap	Aerocyte capillary endothelial cells	Fib	Fibroblasts
Artery	Arterial endothelial cells	Myofib	Myofibroblasts
gCap	General capillary endothelial cells	Fibromyo./SMCs	Fibromyocytes/Smooth muscle cells
Lymph	Lymphatic endothelial cells	Pericyte	Pericyte
Vein	Venous endothelial cells	Neu	Neutrophils
AT1	Alveolar type I cells	MΦ	Macrophages
AT2	Alveolar type II cells	T cells	T cells
Ciliated	Ciliated cells	B cells	B cells
Club	Club cells	Mono.	Monocytes
DC	Dendritic cells		

**FIGURE 3:**
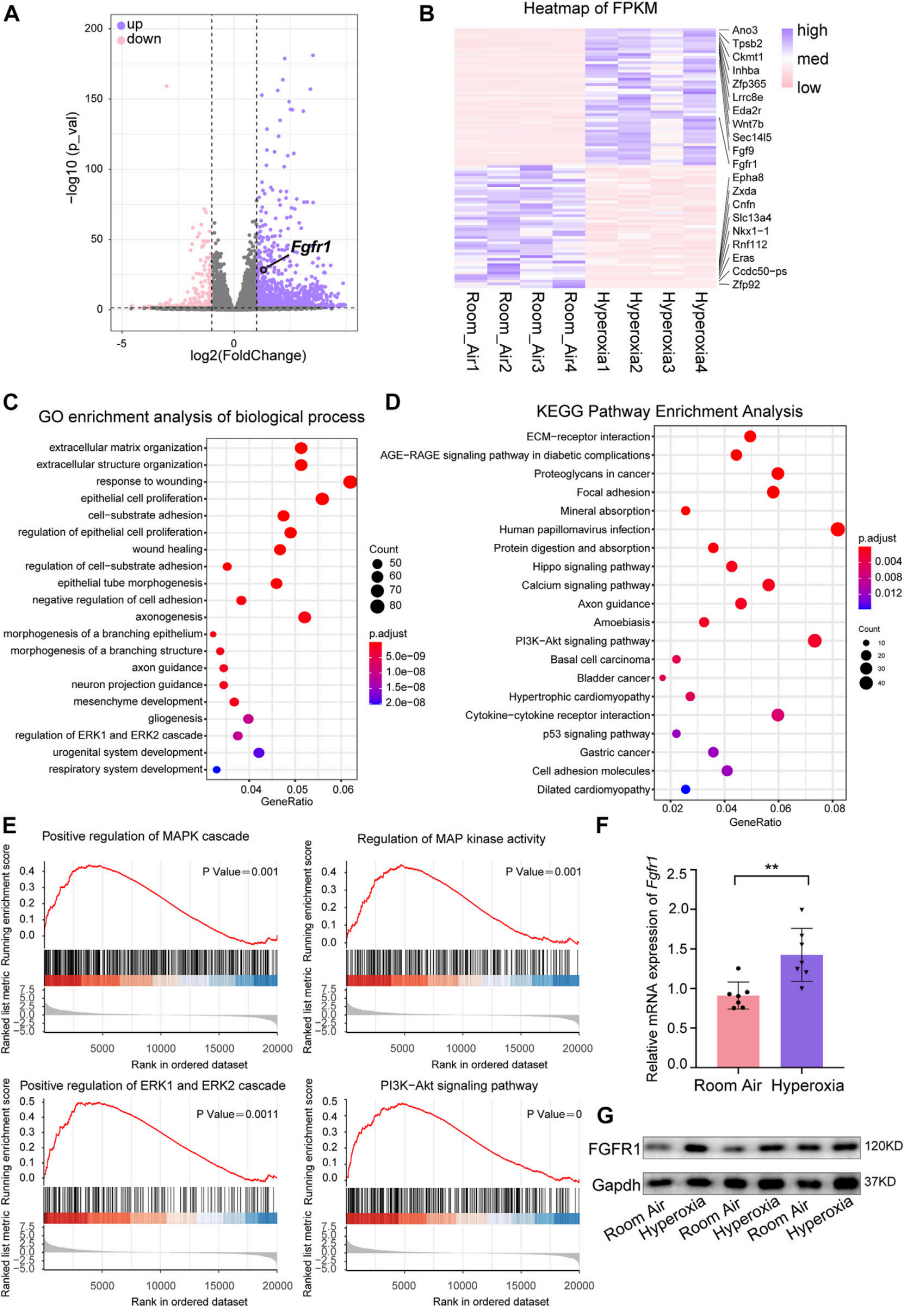
RNA-seq analysis of differentially expressed genes (DEGs) and hyperoxia-impacted signaling pathways in ECs from normal and hyperoxia-impaired lungs. **(A)** Volcano plot showing upregulated and downregulated transcript levels of DEGs, p-value < 0.05, |log2FoldChange|≥1. **(B)** Heatmap of the top 50 upregulated DEGs and top 50 downregulated DEGs. **(C)** Hyperoxia-impacted signaling pathways in ECs as identified by GO enrichment analysis of biological processes. All terms shown are significantly enriched (p-value < 0.05). **(D)** Hyperoxia-impacted signaling pathways in ECs as identified by KEGG pathway enrichment analysis. All terms shown are significantly enriched (p-value < 0.05). **(E)** Upregulated downstream pathways of activated FGFR1 as revealed by Gene Set Enrichment Analysis, all terms shown are significantly enriched (p-value < 0.05). **(F)** qPCR of *Fgfr1* expression in ECs of normoxic or hyperoxic lungs. *n* = 7 per group. Data are shown as means ± SEMs. **p < 0.01. **(G)** Western blot examining the expression of FGFR1 in ECs of normoxic or hyperoxic lung, Gapdh as negative control.

Next, we performed RNA-seq to further verify the key molecular changes in ECs during the occurrence and development of hyperoxia-induced BPD. The results revealed that the expression of endothelial *Fgfr1* was increased in hyperoxic lungs (Figures 3A,B). Furthermore, the enrichment analysis of differentially expressed genes (DEGs) showed that hyperoxia upregulated the downstream signaling pathways of activated FGFR1, including the ERK/MAPK signaling pathway and PI3K-Akt signaling pathway (Figures 3C–E). Notably, hyperoxia also affected the signaling pathways associated with epithelial cell proliferation and epithelial tube morphogenesis (Figure 3C). Consistent with the results of sequencing, the results of qPCR and WB also showed that the expression of FGFR1 was significantly increased in ECs from hyperoxic lungs (Figures 3F,G). We also detected the expression of endothelial FGFR2, another major FGFR expressed by ECs in addition to FGFR1. The results showed that there was no significant difference in endothelial FGFR2 expression between normoxic and hyperoxic neonatal lungs (Supplementary Figures S2A–C). We speculate that this may be attributed to that in neonatal lung, endothelial FGFR1 may be more sensitive to hyperoxia exposure than endothelial FGFR2. These data suggest that hyperoxia upregulates endothelial FGFR1 expression and that the upregulated endothelial FGFR1 may play a vital role in hyperoxia-induced BPD.

### Deficiency of endothelial FGFR1 improves alveologenesis, respiratory function and angiogenesis in hyperoxia-exposed mice

**FIGURE 4:**
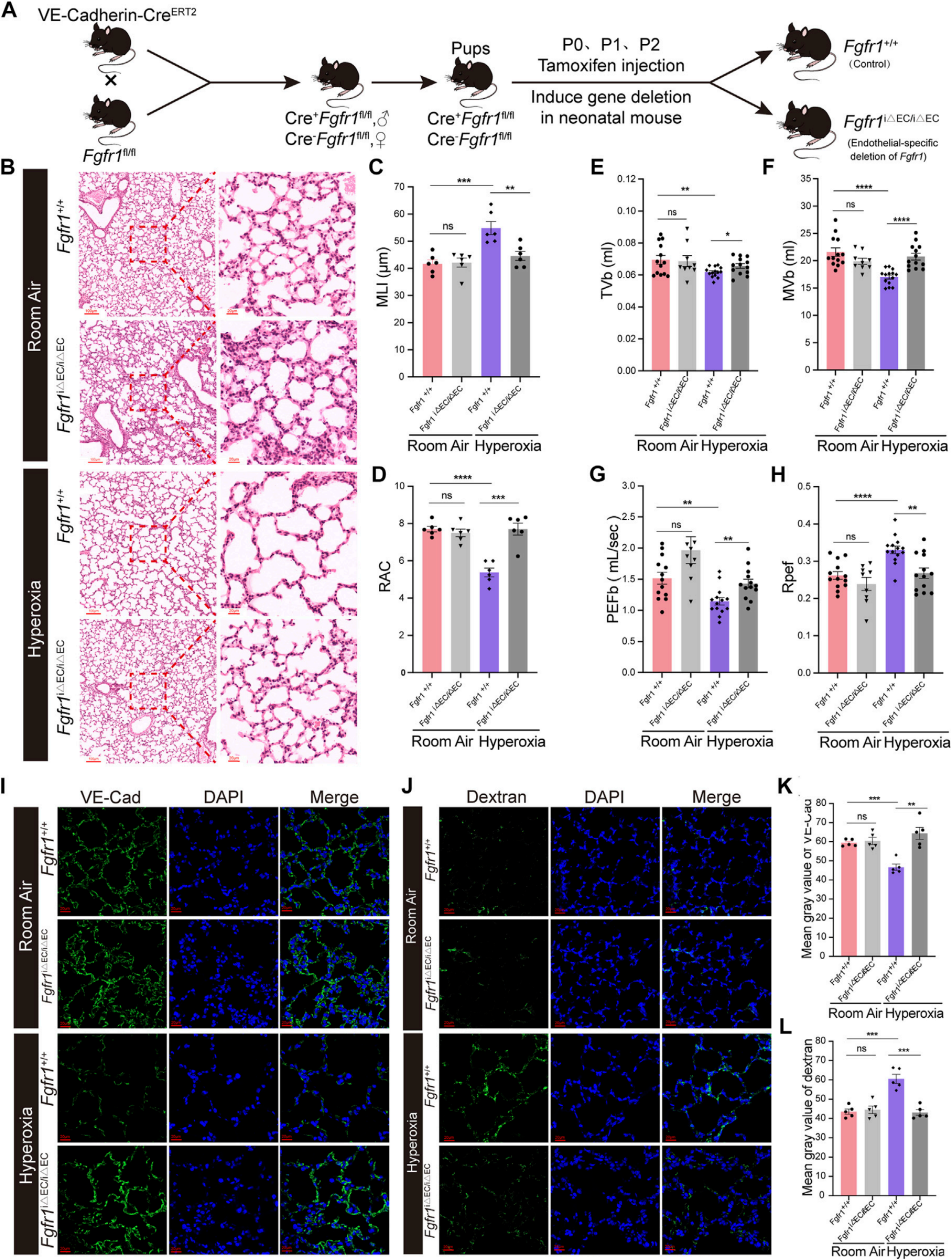
Deletion of endothelial *Fgfr1* improved alveolar development and respiratory metrics and angiogenesis in mice upon hyperoxia. **(A)** Schematic representation of EC-specific inducible deletion of *Fgfr1* in neonatal mice. **(B)** Representative images of H&E-stained lungs from *Fgfr1* ^+/+^ and *Fgfr1* ^iΔEC/iΔEC^ mice reared in room air or hyperoxia. The top panel shows low-magnification (scale bar = 100 μm) images, and the bottom panel shows higher-magnification (scale bar = 20 μm) images. **(C,D)** Quantification of MLI (C) and RAC (D) based on the data in (B). Data are shown as means ± SEMs. *n* = 6 per group. **p < 0.01, ***p < 0.001. **(E–H)** Results of respiratory metrics measurement. Data are shown as means ± SEMs. *n* = 9, 13 or 14 per group. *p < 0.05, **p < 0.01, ****p < 0.0001. **(I)** Representative images of IF staining for VE-Cad in lung sections. Magnification: ×40. Scale bar = 20 μm. **(K)** Mean gray value of VE-Cad based on the data in (I). Data are shown as means ± SEMs. n = 5 per group. **p < 0.01, ***p < 0.001. **(J)** Representative images of FITC-dextran leakage from mice reared in room air or hyperoxia. Magnification: ×40. Scale bar = 20 μm. **(L)** Mean gray value of dextran based on the data in (J). Data are shown as means ± SEMs. *n* = 5 per group. ***p < 0.001.

To dissect the functional contribution of FGFR1 in hyperoxia-induced BPD, we generated the genetically modified mouse in which *Fgfr1* was conditionally deleted specifically in ECs (*Fgfr1* ^iΔEC/iΔEC^) by crossing *Fgfr1* ^fl/fl^ mice with *VE-Cadherin-(PAC)-Cre* ^ERT2^ mice (Figure 4A). The results of qPCR verification showed that the endothelial *Fgfr1* was knockout efficiently (Supplementary Figures S3A–C). Wild-type mice (*Fgfr1* ^+/+^) were used as controls. We found that deletion of *Fgfr1* improved the alveologenesis of neonatal mice upon hyperoxia (Figures 4B–D). After hyperoxia exposure, the MLI of mice from the *Fgfr1* ^iΔEC/iΔEC^ group was significantly decreased, and the RAC was significantly increased compared to mice from the *Fgfr1* ^+/+^ group (Figures 4C,D). Next, we measured the respiratory metrics of *Fgfr1* ^+/+^ and *Fgfr1* ^iΔEC/iΔEC^ mice. Unsurprisingly, deficiency of FGFR1 in ECs improved the respiratory function of neonatal mice upon hyperoxia, with TVb, MVb and PEFb increasing markedly and Rpef decreasing markedly in *Fgfr1* ^iΔEC/iΔEC^ mice (Figures 4E–H). After hyperoxia exposure, the expression of VE-Cad increased in lungs from *Fgfr1* ^iΔEC/iΔEC^ mice compared to that in lungs from *Fgfr1* ^+/+^ mice by detecting with IF staining (Figures 4I,K). The results of FITC-dextran leakage experiment indicated that the vascular leakage of *Fgfr1* ^iΔEC/iΔEC^ mice was decreased in hyperoxia (Figures 4J,L). There were no significant differences observed between normoxia-exposed *Fgfr1* ^+/+^ mice and the normoxia-exposed *Fgfr1* ^iΔEC/iΔEC^ mice or the hyperoxia-exposed *Fgfr1* ^iΔEC/iΔEC^ mice. Here, it is demonstrated that deletion of endothelial *Fgfr1* can protect the lungs from hyperoxia-induced lung injury in neonatal mice.

### Hyperoxia triggers up-expression of FGFR1 in aCap cells rather than in gCap cells

**FIGURE 5:**
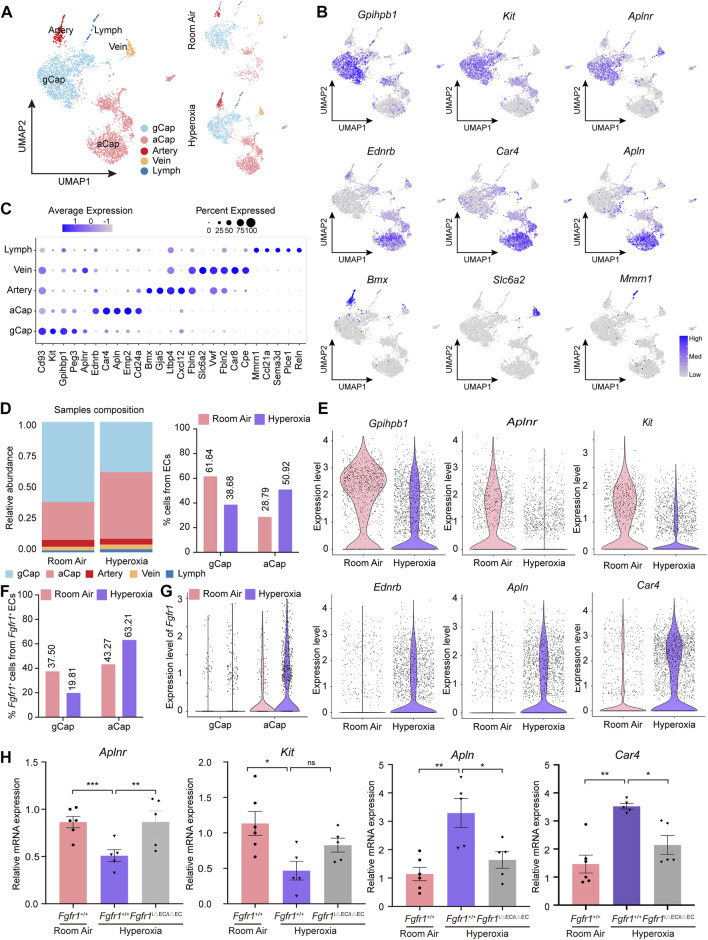
Hyperoxia induced upregulation of FGFR1 mainly in aCap cells rather than in gCap cells. **(A)** A total of 5 clusters of ECs were identified. Cell populations are colored as indicated by the legend. **(B)** Feature plots showing the expression of principal identifiers of general capillary endothelial cells (gCap), aerocyte capillary endothelial cells (aCap), arterial endothelial cells (Artery), venous endothelial cells (Vein) and lymphatic endothelial cells (Lymph). **(C)** Dotplot depicting the top 5 most differentially expressed genes across endothelial clusters. The intensity of expression is indicated as specified by the color legend. **(D)** Alteration of the relative proportion of gCap cells and aCap cells. The right panel shows the cellular compositions of ECs, colored as indicated by the legend, and the left panel shows the relative proportion of gCap cells and aCap cells from ECs. **(E)** Violin plots displaying the expression patterns of gCap cell and aCap cell markers. **(F)** Column chart showing *Fgfr1* ^+^ gCap cells and *Fgfr1* ^+^ aCap cells from total *Fgfr1* ^+^ ECs. **(G)** Violin plots displaying the expression patterns of *Fgfr1* in gCap cells and aCap cells. **(H)** qPCR of gCap cell markers, *Aplnr* and *Kit* expression and aCap cell markers, *Apln* and *Car4* expression in ECs of lung from *Fgfr1* ^+/+^ and *Fgfr1* ^iΔEC/iΔEC^ mice reared in room air or hyperoxia. *n* = 5 or 6 per group. Data are shown as means ± SEMs. ^ns^no significant, *p < 0.05, **p < 0.01, ***p < 0.001.

To shed light on the underlying regulatory mechanisms of endothelial FGFR1 in hyperoxia-induced lung injury, we analyzed the alteration of EC subpopulations by scRNA-seq. Five distinct EC subpopulations, including gCap, aCap, Artery, Vein and Lymph, were identified based on their expression profiles (Figures 5A–C; Table 1). Hyperoxia reduced the frequency of gCap cells while increasing the frequency of aCap cells, which is consistent with the results obtained by analyzing the raw data from Thébaud (Figure 5D, Supplementary Figures S4D–E). Corresponding to the changes in cell proportion, in hyperoxia-impaired lung, the expression of gCap cell markers, *Gpihpb1*, *Aplnr* and *Kit* were decreased, while the expression of aCap cell markers, *Ednrb*, *Apln* and *Car4* were increased (Figure 5E).

To determine whether FGFR1 results in these changes, we first analyzed the expression patterns of *Fgfr1* in gCap cells and aCap cells. Interestingly, hyperoxia increased the frequency of *Fgfr1* ^+^ aCap cells, and *Fgfr1* ^+^ aCap cells accounted for the majority of *Fgfr1* ^+^ ECs in hyperoxia (Figure 5F). Additionally, the increment of *Fgfr1* expression was mainly observed in aCap cells rather than in gCap cells (Figure 5G). Next, we found that in hyperoxic lung ECs, *Fgfr1* ^iΔEC/iΔEC^ mutant increased the mRNA expression of gCap cell marker, *Aplnr*, while decreased the mRNA expression of aCap cell markers, *Apln* and *Car4* (Figure 5H). These data indicate that FGFR1 may be pivotal in maintaining the proportion and cellular function of gCap cells and aCap cells upon hyperoxia.

### Inhibition of endothelial FGFR1 ameliorates hyperoxia-induced alveolar damage and respiratory dysfunction

**FIGURE 6:**
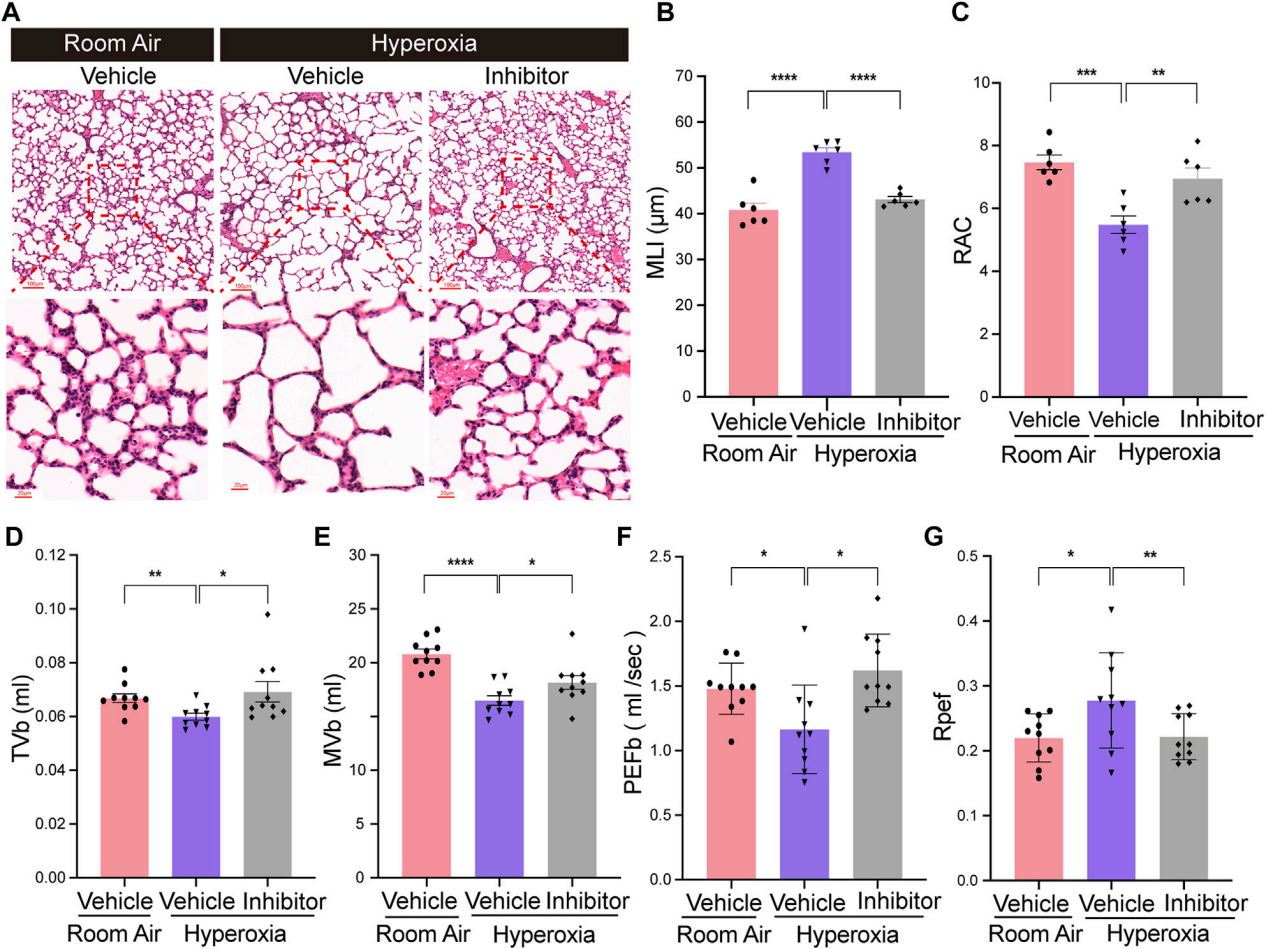
Inhibition of endothelial Fgfr1 improved alveolar development and respiratory metrics in neonatal mice in hyperoxia. **(A)** Representative images of H&E-stained lungs from vehicle or FGFR1 inhibitor treated mice. The top panel shows low-magnification (scale bar = 100 μm) images, and the bottom panel shows higher-magnification (scale bar = 20 μm) images. **(B,C)** Quantification of MLI (B) and RAC (C) based on the data in (A). Data are shown as means ± SEMs. n = 6 per group. **p < 0.01, ***p < 0.001, ****p < 0.0001. **(D–G)** Results of respiratory metrics measurement. Data are shown as means ± SEMs. *n* = 10 per group. *p < 0.05, **p < 0.01, ****p < 0.0001.

To verify whether FGFR1 could be a potential therapeutic target for BPD, the effects of the FGFR1 inhibitor (AZD4547) on hyperoxia-induced BPD was evaluated. The results showed that inhibition of FGFR1 by AZD4547 also improved the alveologenesis of neonatal mice upon hyperoxia (Figure 6A). AZD4547 treatment significantly decreased the MLI of hyperoxia-exposed mice, while significantly increased the RAC of hyperoxia-exposed mice (Figures 6B,C). In addition, the results of respiratory metrics measurement showed that after hyperoxia exposure, mice from AZD4547 treated group had increased TVb, MVb and PEFb, while had decreased Rpef (Figures 6D–G). Consistent with the results of the *Fgfr1* genetic deficiency model, these data demonstrated that FGFR1 may be a potential therapeutic target for BPD.

## Discussion

Oxygen supplementation is the most common treatment in newborns. However, hyperoxic damage is one of the main factors that blunts normal lung alveologenesis and the development of pulmonary microvasculature. In this work, neonatal mice exposed to hyperoxia ultimately exhibited simplified alveolarization and enlarged alveolar cavity, the prominent features of BPD. Additionally, we reported a decrease in MVb and TVb in mice with BPD, which indicates the destruction of respiratory function in mice. The worsening of PEFb and Rpef, reference indicators of airflow restriction, perhaps suggested decreased respiratory muscle strength and increased airway obstruction (Menachery et al., 2015; Ramírez-Ramírez et al., 2017). These data suggest that neonatal hyperoxia exposure disrupted alveologenesis and ultimately led to obstructed respiratory function, which indicated that we established the hyperoxia-induced BPD mouse model successfully and stably.

We observed that hyperoxic mice exhibited abnormal vascular development and mice from hyperoxia group had reduced vascular branching and density. Additionally, the increased leakage of vasculature also suggested that hyperoxia disrupted blood vessels. Disruption of lung angiogenesis is a key mechanism through which hyperoxia leads to alveolar simplification in BPD (Ren et al., 2019). Previous studies have shown that ECs play a critical role in organ injury and repair (Cao et al., 2016; Gladka et al., 2021; Li et al., 2022), so does in hyperoxia-induced lung injury. It was reported that hyperoxia specifically decreased fatty acid oxidation (FAO) in ECs, which induced apoptosis and simplified alveolarization and vascularization in the lungs of mice (Yao et al., 2019). Exposure of human microvascular endothelial cells to hyperoxia decreased cell viability and proliferation (Attaye et al., 2017). Moreover, it was previously found that hyperoxia could evoke mitochondrial DNA damage and mitochondrial fragmentation in pulmonary endothelial cells, which would result in pulmonary endothelial cell dysfunction (Ma et al., 2018). These data demonstrateed that ECs are crucial to the hyperoxia-induced lung injury response.

We further investigated the cellular and molecular changes resulting from hyperoxia-induced lung injury by performing scRNA-seq analysis of normal and hyperoxia-injured lungs and RNA-seq analysis of ECs in lungs from normal mice and hyperoxia-exposed BPD mice. We reported that hyperoxia decreased the proportion of ECs in lungs of neonatal mice. FGFR1 was upregulated in ECs of the hyperoxia-damaged lungs, which was also confirmed in the results of qPCR and WB. Nevertheless, we did not observe significant difference in the expression of endothelial FGFR2. We hypothesized that perhaps endothelial FGFR1 was more sensitive to hyperoxia exposure than endothelial FGFR2. A previous study showed that FGFR1 and FGFR2 were the dominant FGFRs in ECs (Presta et al., 2005) and were autophosphorylated and activated upon the binding of FGF ligands, such as FGF1 and FGF2 (Goetz and Mohammadi, 2013; Xie et al., 2020). In some studies, endothelial FGF/FGFR signals have been reported as facilitative for angiogenesis under physiological or pathological states (Tsuboi et al., 1992; Yanagisawa-Miwa et al., 1992; Compagni et al., 2000; Murakami and Simons, 2009; De Smet et al., 2014). Nevertheless, other findings reported that FGF/FGFR signaling is not essential for angiogenesis. Mice with deficiency of FGF1 or FGF2, even deficiency of FGF1 and FGF2 together had few angiogenesis abnormalities (Ortega et al., 1998; Zhou et al., 1998; Miller et al., 2000). Moreover, there is another point of view that endothelial FGFR1 and FGFR2 are necessary for pathological neovascularization after injury but not for physiological vascular development and vascular homeostasis (Oladipupo et al., 2014; House et al., 2016). Interestingly, studies reported that overactivated FGFR1 could suppress angiogenesis and promote fibrosis. A previous study reported that exaggerated endothelial FGF2/FGFR1 signaling caused by SUMOylation-defective mutation of FGFR1 suppressed VEGFA/VEGFR2 signaling and the angiogenic capabilities of ECs (Zhu et al., 2022). In another study, it was reported that overactivation of FGFR1 contributed to the occurrence of fibrosis (Ding et al., 2014), a prominent feature of severe BPD. The present study, as well as previous studies, demonstrated that in different situations, balancing the level of FGFR1 is important for physiological or pathological activities. The upregulation of the downstream signaling pathways of activated FGFR1 further demonstrated that hyperoxia activated FGFR1 signaling pathways. These data suggested that hyperoxia-induced upregulation of endothelial FGFR1 expression may be a key factor in hyperoxia-induced abnormal vascular development and ultimately result in interrupted alveologenesis and pulmonary dysfunction.

Therefore, we treated the *Fgfr1* ^iΔEC/iΔEC^ mice with the same hyperoxia exposure protocol to further address the role of FGFR1 in hyperoxia-induced lung injury and repair. We found that deletion of endothelial *Fgfr1* protected the lungs from hyperoxia-induced lung injury in neonatal mice. After hyperoxia exposure, mice from *Fgfr1* ^iΔEC/iΔEC^ group had improved angiogenesis, alveologenesis and respiratory metrics. The results of scRNA-seq analysis suggested that hyperoxia decreased the frequency of gCap cells but increased the frequency of aCap cells. Consistently, downregulation of gCap cell markers and upregulation of aCap cell markers were detected in hyperoxic lungs. The *c-Kit* ^+^ endothelial cells (gCap) were reported to be capillary progenitors, which could enhance lung angiogenesis and prevent alveolar simplification in neonatal mice exposed to hyperoxia (Miranda et al., 2013; Ren et al., 2019). In addition, gCap cells were shown to be critical for normal neonatal lung angiogenesis (Wang et al., 2022). Thus, it makes sense to believe that the reduction in gCap cells caused by hyperoxia may be crucial to disrupted vascular development in newborn mice. On the other hand, previously published data showed that the number of aCap cells increased after hyperoxia exposure, accompanied by upregulation of inflammatory genes and antiangiogenic genes, which contribute to BPD (Hurskainen et al., 2021). The other point of view supports that *Car4* ^+^ capillary endothelial cells (aCap) are important for normal alveolar development by interacting with AT1 cells through VEGFA and contribute to alveolar revascularization postinjury (Niethamer et al., 2020; Vila Ellis et al., 2020). Consequently, we hypothesize that the hyperoxia-induced increase in aCap cells resulting in upregulation of the expression of some unfriendly genes (such as inflammatory genes and antiangiogenic genes), through which aCap cells interact with other cells (such as AT1 cells), may be a major factor in disrupting lung development and regeneration in neonatal mice.

Interestingly, scRNA-seq analysis revealed that the upregulated expression of endothelial *Fgfr1* was mainly found in aCap cells rather than in gCap cells. We validated the correlation of endothelial FGFR1 deficiency with the expression of gCap cell markers and aCap cell markers upon hyperoxia. We observed that deletion of *Fgfr1* increased the expression of gCap cell markers but decreased the expression of aCap cell markers in hyperoxic lung ECs. Thus, we speculate that the upregulated FGFR1 in aCap may affect the cellular proportion and function of gCap cells and aCap cells upon hyperoxia. In the future, further researches are needed to determine their regulatory relationships and the underlying mechanisms.

Finally, to mimic the clinical treatment method, we treated neonatal mice with FGFR1 inhibitor by intragastric injection of AZD4547 to induce FGFR1 deficiency. Consistent with the results of the *Fgfr1* genetic deficiency model, inhibition of FGFR1 by AZD4547 also showed protective function against hyperoxia-induced lung injury in neonatal mice. After hyperoxia exposure, mice from inhibitor treated group had improved alveologenesis and respiratory metrics. These data suggest that FGFR1 may be a potential therapeutic target for BPD.

In summary, the present study shows that hyperoxia upregulates the expression of FGFR1 and FGFR1 signaling pathways in lung ECs of neonatal mice. Deletion of endothelial *Fgfr1* can protect lung from hyperoxia-induced lung injury in neonatal mice, which may be attributed to the regulation of FGFR1 on gCap cells and aCap cells. The results of the present research suggest that FGFR1 is a potential therapeutic target for BPD, which has considerable clinical value.

## Data Availability

All sequencing data, including raw fastq sequencing files, gene expression matrices, and associated metadata generated in this study have been deposited in the NCBI’s Gene Expression Omnibus (GEO) database under accession number GSE217489 (https://www.ncbi.nlm.nih.gov/geo/query/acc.cgi?acc?GSE217489). Further inquiries can be directed to the corresponding author.
