# Supplementary material for: Deficiency of endothelial FGFR1 alleviates hyperoxia-induced bronchopulmonary dysplasia in neonatal mice
Source: Front Pharmacol. 2022 Nov 18;13:1039103. doi: 10.3389/fphar.2022.1039103 (PMC9716472; doi:10.3389/fphar.2022.1039103)
Supplement: Supplementary file 1 [file DataSheet1.ZIP › Supplementary_Material/Supplementary_Material.pdf]

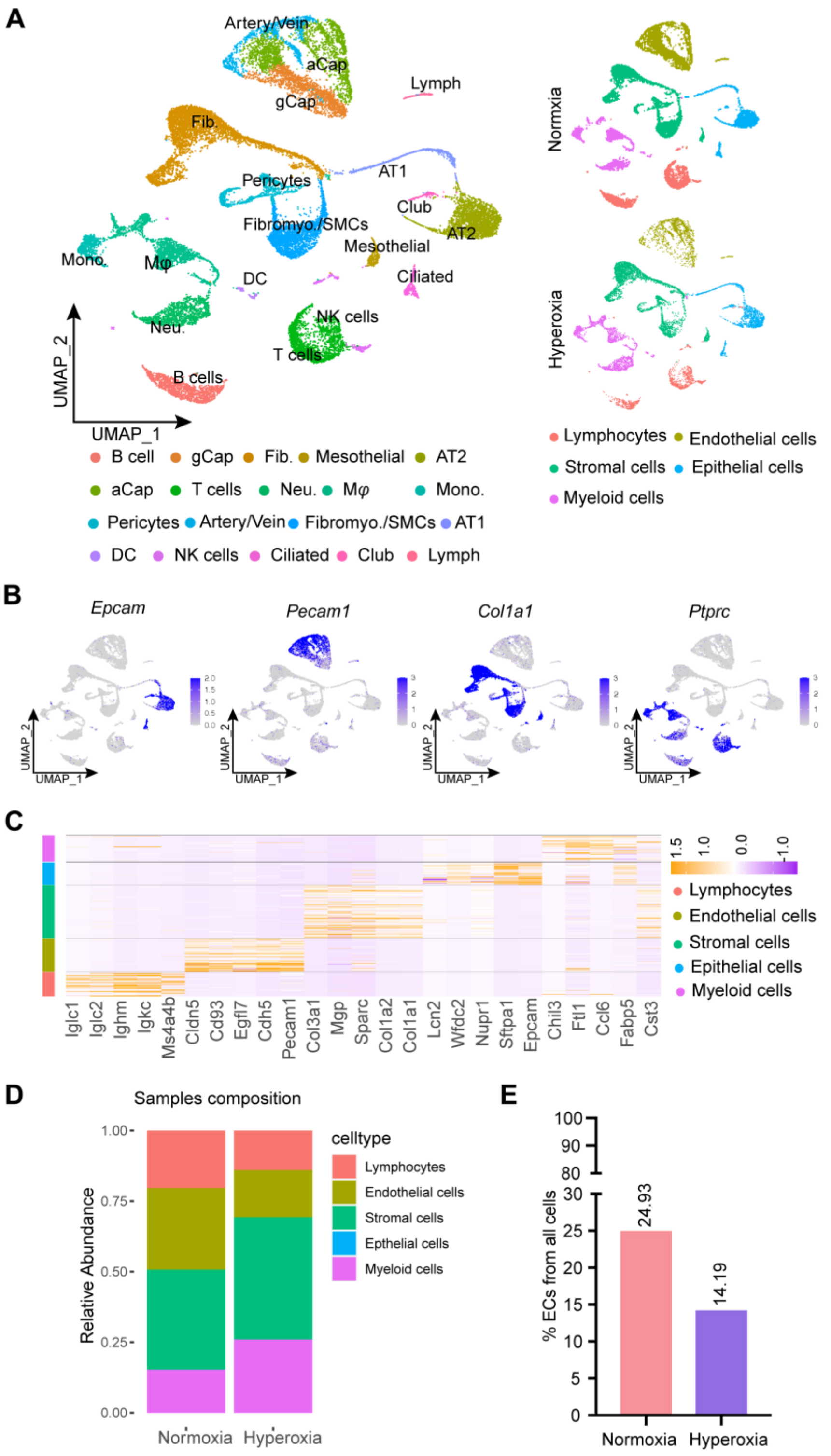

Figure s1. Hyperoxia decreased the frequency of endothelial cells.

(**A**) UMAP plot of all scRNA-seq data analyzing from the raw data published by Thébaud et al. showing a total of 20 distinct cell types corresponding to 5 major cell groups. Cell populations are colored as indicated by the legend.

(**B**) Feature plots showing the expression of principal identifiers of epithelial cells, endothelial cells, stromal cells and immune cells populations.
(**C**) Heatmap of the top differentially expressed genes across 5 major cell types. The intensity of expression is indicated as specified by the color legend.
(**D**) Cellular compositions are colored as indicated by the legend in normal and hyperoxia-impaired lungs.
(**E**) The relative proportion of endothelial cells from all cells in normal and hyperoxia-impaired lungs.

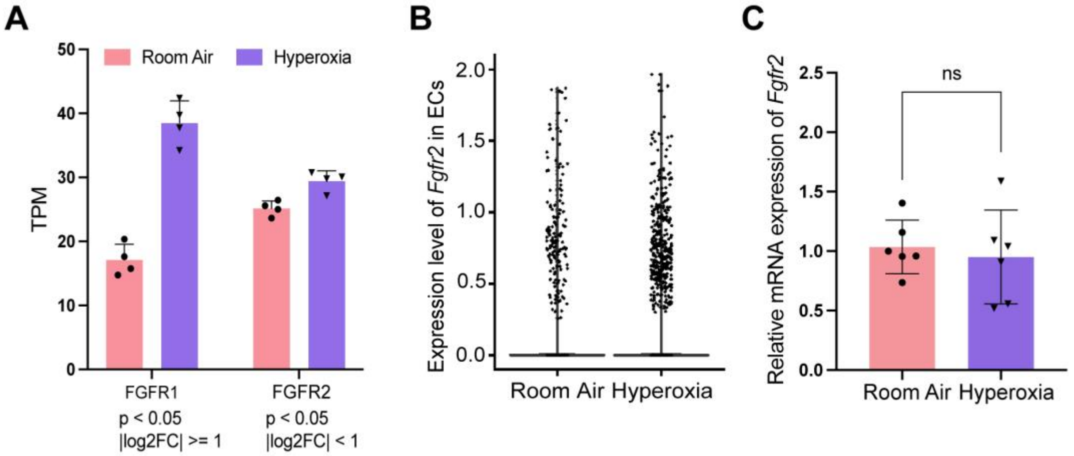

Figure s2. There was not significant difference in the expression of endothelial *Fgfr2* between hyperoxic lung and normoxic lung.
(A) RNA-seq analysis of DEGs and TPM value of *Fgfr1* and *Fgfr2* . The analysis of DEGs showed hyperoxia increased the expression of endothelial *Fgfr1* significantly, but have no significant effect on endothelial *Fgfr2* expression. Genes with p value < 0.05, |log2FoldChange| >= 1 were identified as significant DEGs.
(B) Violin plot showing the expression pf *Fgfr2* in ECs.
(C) qPCR of *Fgfr2* expression in ECs of normoxic or hyperoxic lung. n = 6 per group. Data are shown as means ± SEMs. [ns]no significant.

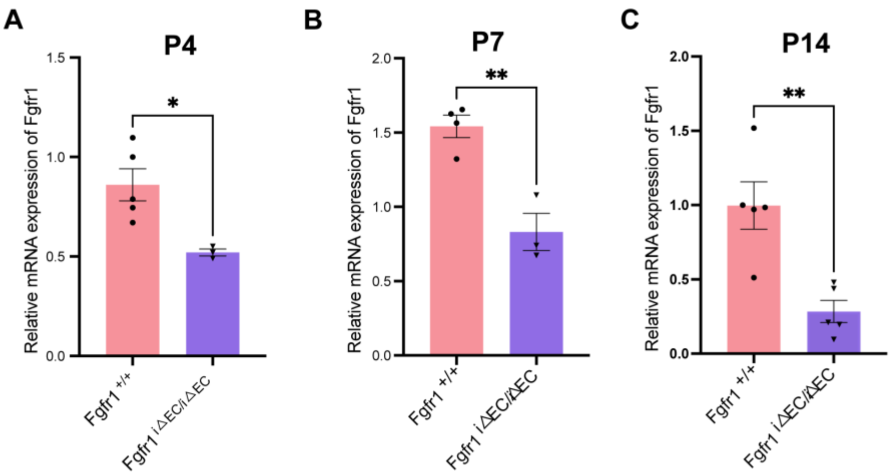

Figure s3. RT-qPCR results demonstrated efficient knockout of *Fgfr1* expression in lung ECs of neonatal mice.

(A) qPCR of *Fgfr1* expression in lung ECs from *Fgfr1* $^{+/+}$ and *Fgfr1*$^{i\Delta EC/i\Delta EC}$ mice at P4.

(B) qPCR of *Fgfr1* expression in lung ECs from *Fgfr1* $^{+/+}$ and *Fgfr1*$^{i\Delta EC/i\Delta EC}$ mice at P7.

(C) qPCR of *Fgfr1* expression in lung ECs from *Fgfr1* $^{+/+}$ and *Fgfr1*$^{i\Delta EC/i\Delta EC}$ mice at P14.

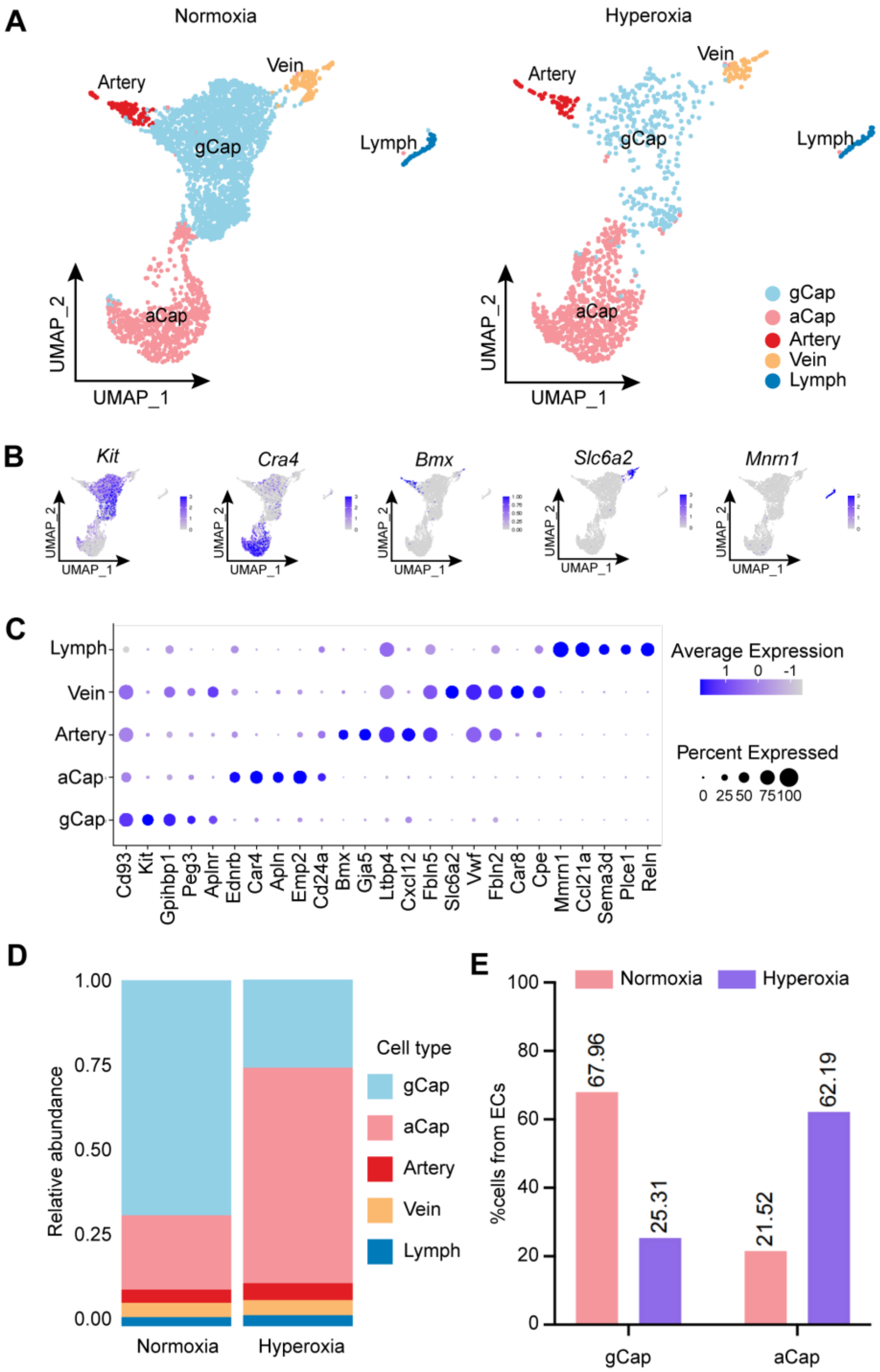

Figure s4. Hyperoxia altered the relative proportion of gCap and aCap.

(**A**) UMAP plot of scRNA-seq data analyzing from the raw data published by Thébaud et al. showing a total of 5 EC clusters identified. Cell populations are colored as indicated by the legend.

(**B**) Feature plots showing the expression of principal identifiers of general capillary endothelial cells (gCap), aerocyte capillary endothelial cells (aCap), arterial endothelial cells (Artery), venous endothelial cells (Vein) and lymphatic endothelial cells (Lymph).

(**C**) Heatmap of the top 5 differentially expressed genes across endothelial clusters. The intensity of expression is indicated as specified by the color legend.

(**D-E**) Alteration of the relative proportion of gCap cells and aCap cells in lungs form normxia and hyperoxia groups. The cellular compositions of ECs (D), colored as indicated by the legend. The relative proportion of gCap cells and aCap cells from ECs (E).
