# Supplementary figures and images for: Deficiency of endothelial FGFR1 alleviates hyperoxia-induced bronchopulmonary dysplasia in neonatal mice
Source: Front Pharmacol. 2022 Nov 18;13:1039103. doi: 10.3389/fphar.2022.1039103 (PMC9716472; doi:10.3389/fphar.2022.1039103)

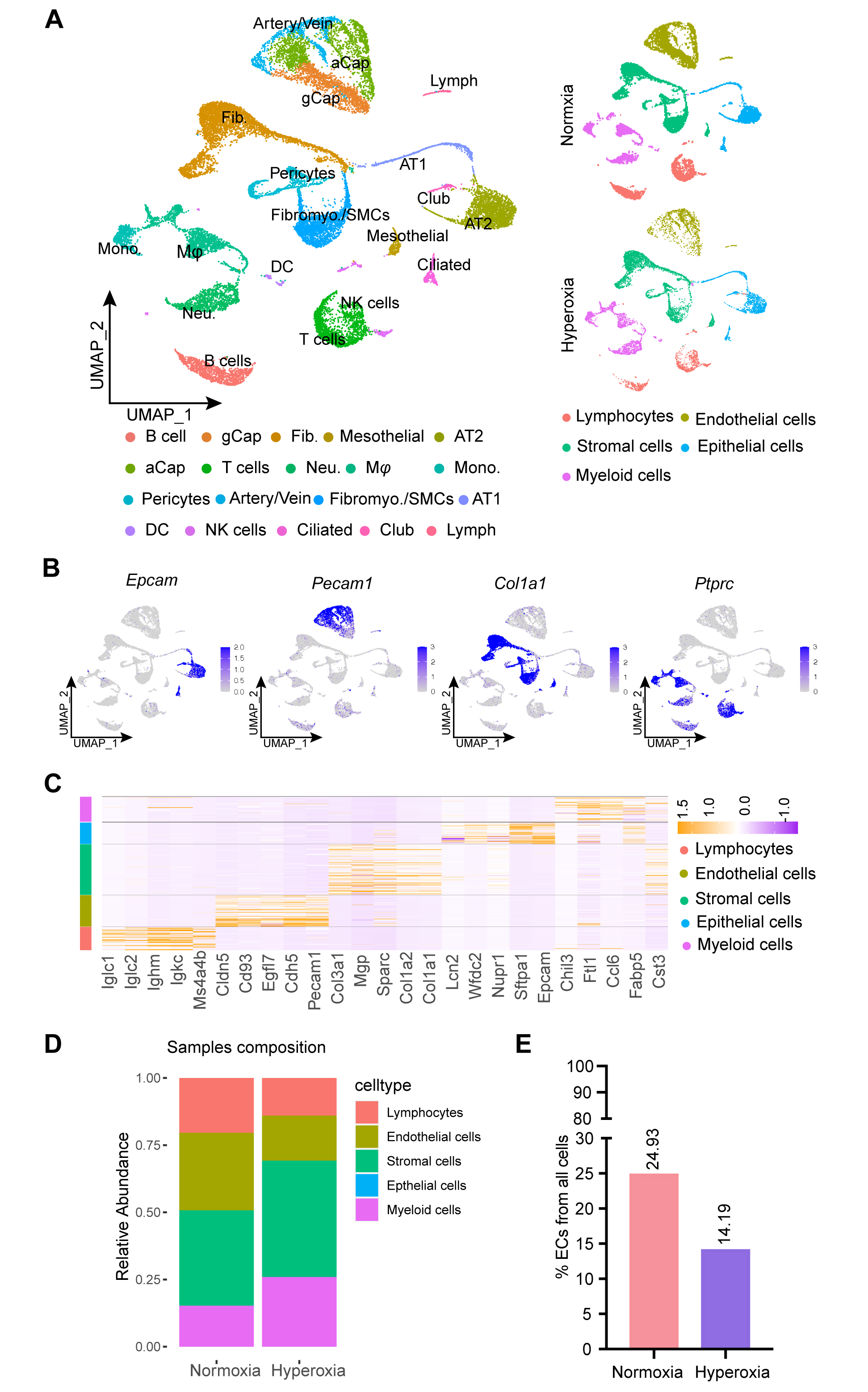

Supplement: Supplementary file 1 [file DataSheet1.ZIP › Supplementary_Material/Figure s1.tif]

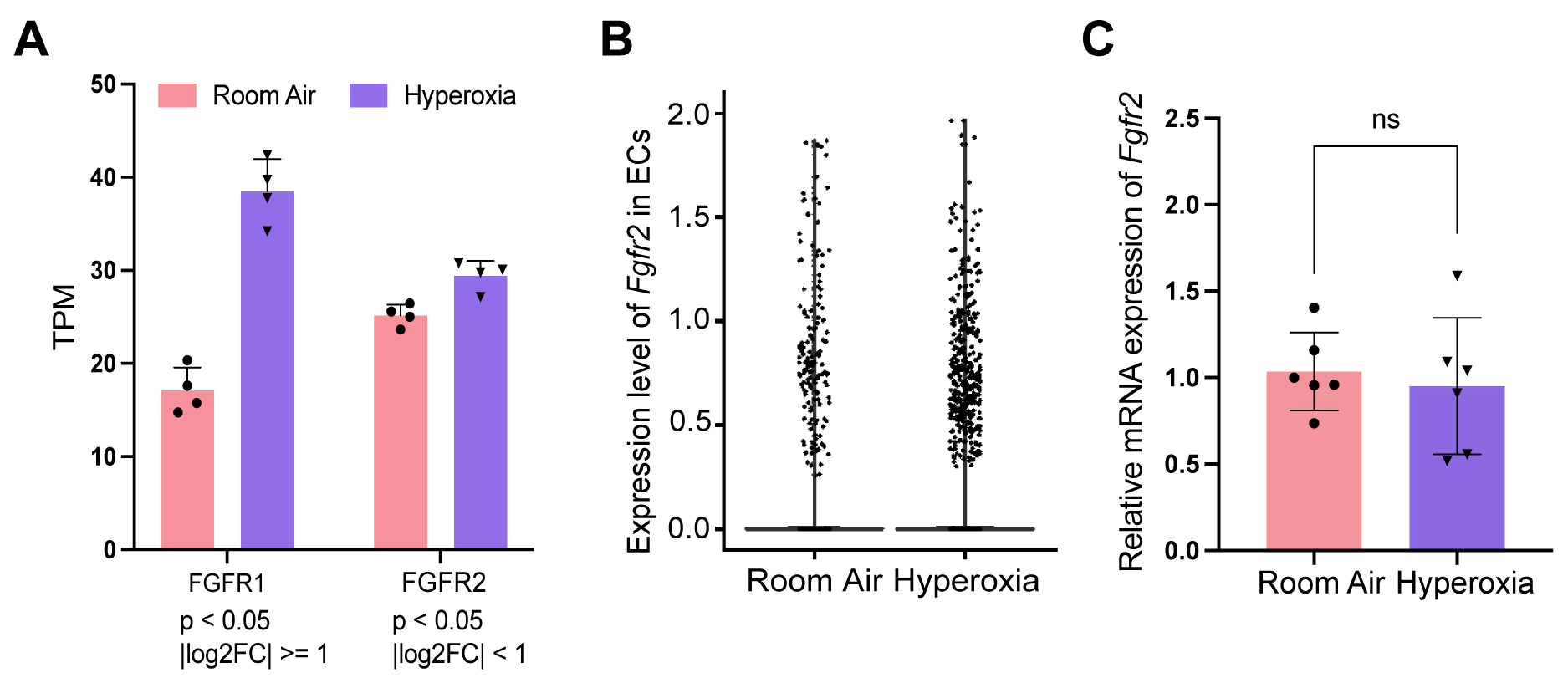

Supplement: Supplementary file 1 [file DataSheet1.ZIP › Supplementary_Material/Figure s2.tif]

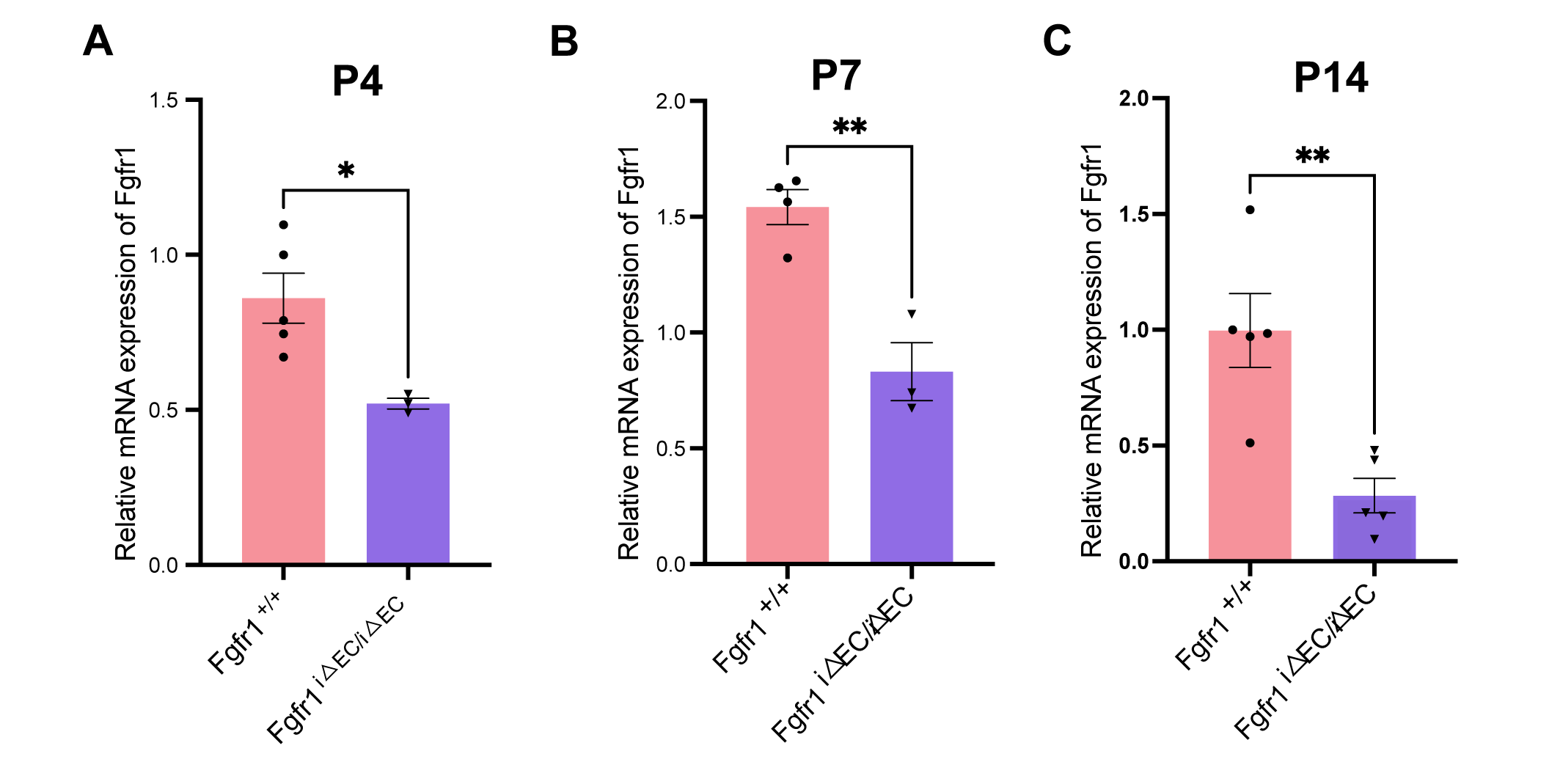

Supplement: Supplementary file 1 [file DataSheet1.ZIP › Supplementary_Material/Figure s3.tif]

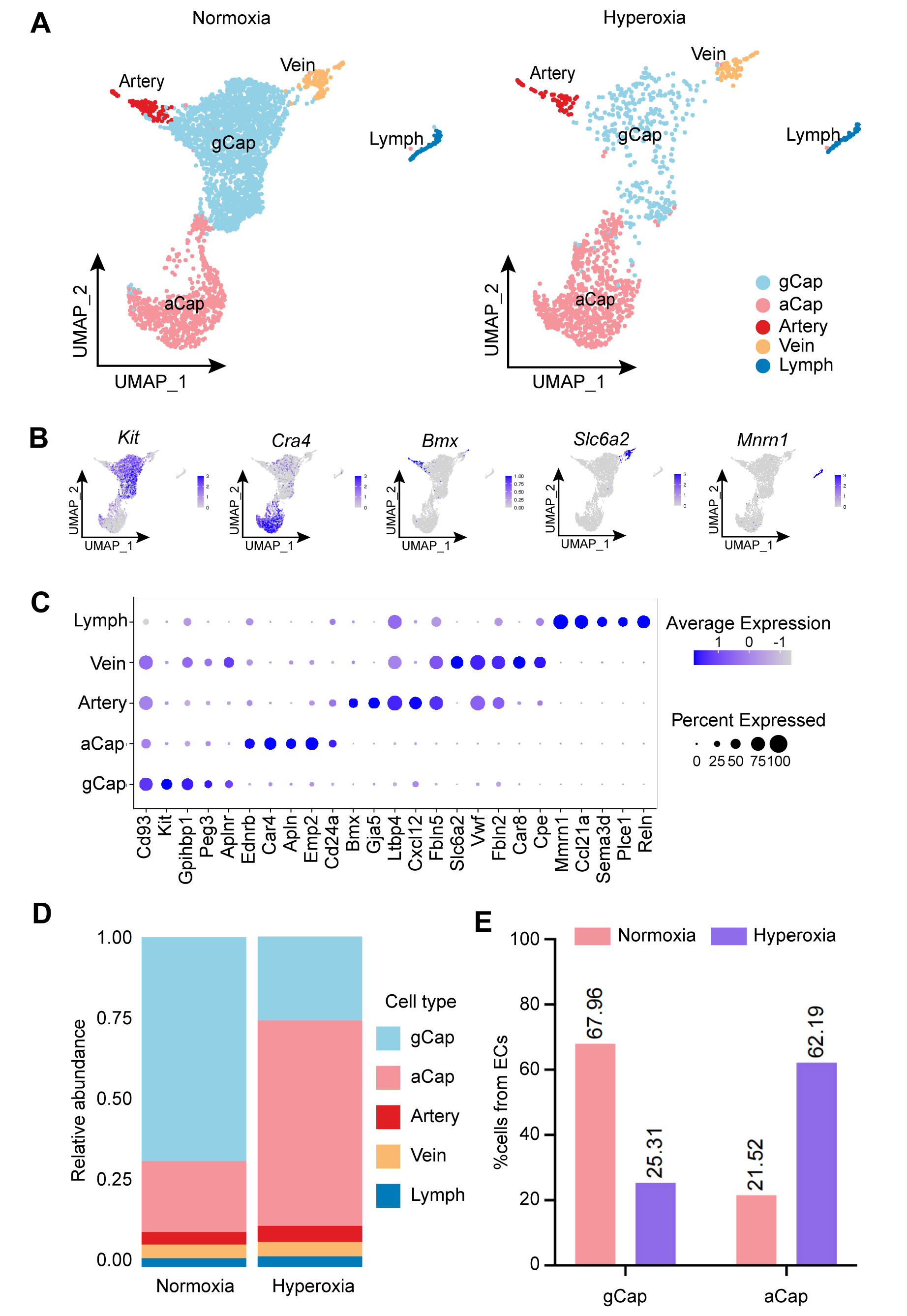

Supplement: Supplementary file 1 [file DataSheet1.ZIP › Supplementary_Material/Figure s4.tif]
